# Supplementary material for: The causal relationship between the human gut microbiota and pyogenic arthritis: a Mendelian randomization study
Source: Front Cell Infect Microbiol. 2024 Nov 26;14:1452480. doi: 10.3389/fcimb.2024.1452480 (PMC11629706; doi:10.3389/fcimb.2024.1452480)
Supplement: Supplementary file 1 [file DataSheet1.zip › mendelian test/ebi-a-GCST90027602.csv_forest.pdf]

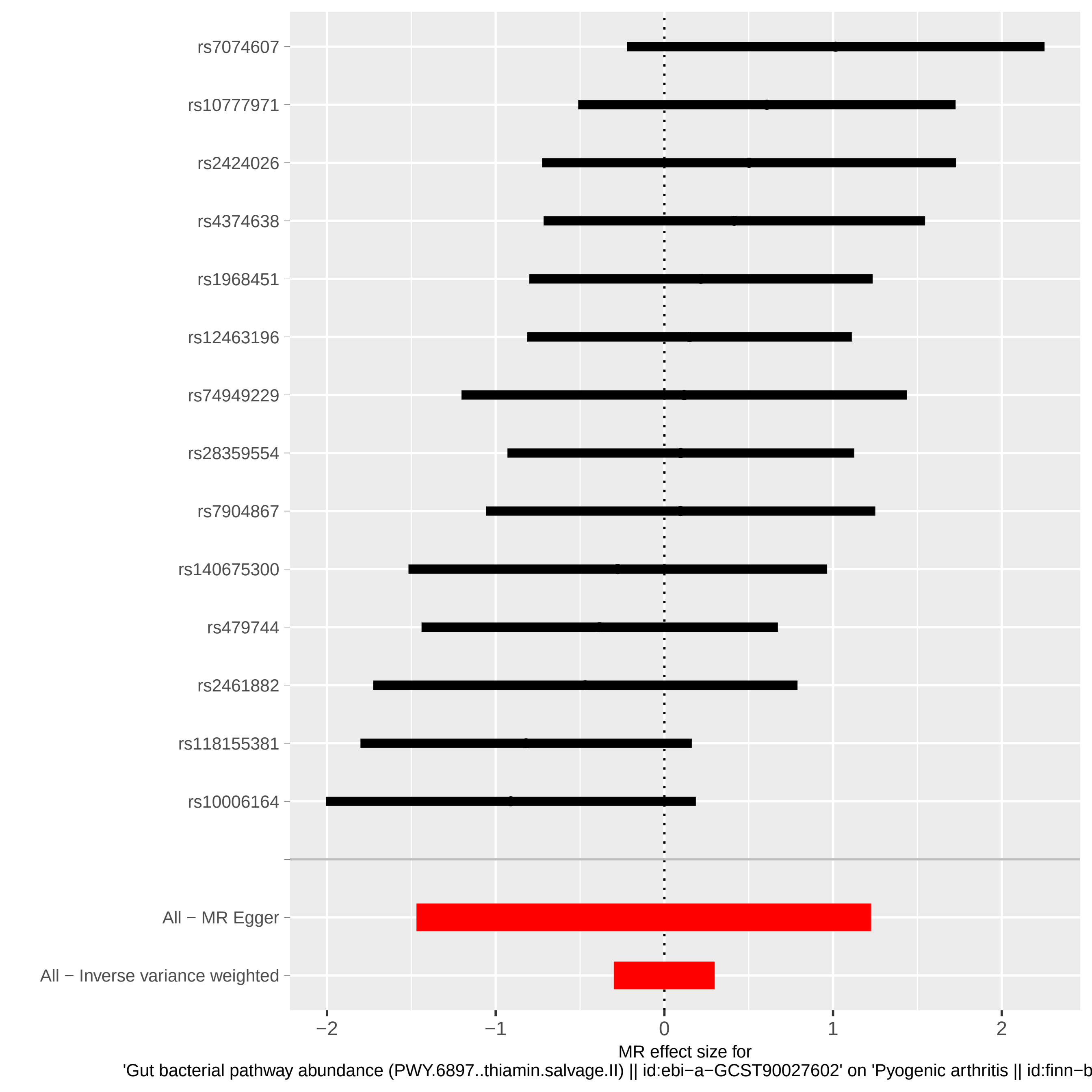

rs7074607
rs10777971
rs2424026
rs4374638
rs1968451
rs12463196
rs74949229
rs28359554
rs7904867
rs140675300
rs479744
rs2461882
rs118155381
rs10006164
All − MR Egger
All − Inverse variance weighted
−2
−1
0
1
2
MR effect size for
'Gut bacterial pathway abundance (PWY.6897..thiamin.salvage.II) || id:ebi−a−GCST90027602' on 'Pyogenic arthritis || id:finn−b
